# Effects of Astragalus Extract Mixture HT042 on Circulating IGF-1 Level and Growth Hormone Axis in Rats

**DOI:** 10.3390/children8110975

**Published:** 2021-10-28

**Authors:** Donghun Lee, Byung-Hak Kim, Sung-Hyun Lee, Won-Young Cho, Young-Sik Kim, Hocheol Kim

**Affiliations:** 1 Department of Herbal Pharmacology, College of Korean Medicine, Gachon University, 1342 Seongnamdae-ro, Sujeong-gu, Gyeonggi-do, Seongnam-si 13120, Korea; dlee@gachon.ac.kr (D.L.); ready1838@naver.com (W.-Y.C.); 2 Korea Institute of Science and Technology for Eastern Medicine (KISTEM) NeuMed Inc., 88 Imun-ro, Dongdaemun-gu, Seoul 02440, Korea; protein0826@snu.ac.kr (B.-H.K.); lsh@neumed.co.kr (S.-H.L.); 3 Department of Herbology, College of Korean Medicine, Woosuk University, 61, Seonneomeo 3-gil, Wansan-gu, Jeollabuk-do, Jeonju-si 55338, Korea; yjbsik@woosuk.ac.kr; 4 Department of Herbal Pharmacology, College of Korean Medicine, Kyung Hee University, 26 Kyungheedae-ro, Dongdaemun-gu, Seoul 02447, Korea

**Keywords:** HT042, circulating insulin-like growth factor-1, growth hormone, growth plate, immunohistochemistry

## Abstract

*Astragalus* extract mixture HT042 is a standardized functional food granted by the Korean FDA for promoting “Children’s Height Growth”. In this study, we determined whether HT042 affects circulatory Insulin-like growth factor-1 (IGF-1) after administration and investigated whether Growth hormone (GH), Growth hormone-releasing hormone receptor (GHRH-R), and Growth hormone secretagogue receptor (GHS-R) mRNAs are expressed in the pituitary, and whether Growth hormone-releasing hormone (GHRH) and Somatostatin (SST) are expressed in the hypothalamus. We also evaluated the growth effect of HT042 on endochondral bone formation. Male Sprague-Dawley rats in the control and HT042 groups were orally administered a single dose of the control and HT042, respectively, and those in the recombinant human GH (rhGH) group were subcutaneously injected with rhGH. Tetracycline was injected intraperitoneally 72 h prior to sacrifice to decide endochondral bone formation. To determine the endocrine or paracrine/autocrine mechanism, we evaluated the expression of local BMP-2 and IGF-1, an immunohistochemical study after HT042 administration. It was confirmed that the growth-promoting effect of HT042 can be contributed to the increase in serum IGF-1, which can be stimulated by GH secretion. Administration of HT042 modulated the activity of GHRH-R and GHR-S in the pituitary gland and promoted GH secretion, thereby changing longitudinal growth through GH/IGF-1 mediation. Results for GHRH and SST expression demonstrated that the hypothalamus can be influenced and mediated by HT042 through a complex neuroendocrine regulatory system. In addition, it was confirmed by oral administration for 10 days that HT042 increased bone formation in cartilage, which is important for height growth. The effect of HT042 could be owing to upregulation of local Bone morphogenetic protein-2 (BMP-2) and IGF-1 expression in the growth plate, which could be regarded as a GH-dependent autocrine/paracrine pathway, as well as circulatory IGF-1.

## 1. Introduction

Growth and development are achieved by adequate nutritional supply externally and by a combination of hormones internally, and if any of these external and internal mechanisms of action are functioning abnormally, normal growth cannot be expected [1].

Long bone growth is a consequence of endochondral ossification process in which cartilage changes to bone, mainly controlled by growth hormone (GH) and insulin-like growth factor-1 (IGF-1) [2]. GH plays a chief role in postnatal growth, which is secreted in a pulsating manner in the anterior pituitary gland [3]. This pulsatile secretion plays an important part in metabolic action, which is induced by growth hormone-releasing hormone (GHRH) and is inhibited by somatostatin (SST) [4]. In addition, GH regulates growth either directly through receptors or indirectly by inducing liver or local production through IGH-1 [5].

IGF-1 is an endocrine hormone in the ternary form combined to IGF binding protein-3 (IGFBP-3) and acid-labile subunit (ALS). Although it is produced in most tissues, it is synthesized by GH and is mainly secreted by the liver [6]. IGF-1 is an active substance that promotes growth in serum, exerts metabolic effects such as insulin production, and is involved in tissue growth and development [7]. In addition to hormonal action, IGF-I shows local action through paracrine and autocrine pathways. In particular, it plays the role of a growth factor that induces the proliferation of bone, muscle, and cartilage [8].

Since the secretion of GH is through pulsatile release, the hormonal levels vary with measurement time, and the difference between these levels can go up to 40 µg/L. For this reason, the serum IGF-1 level is considered as superior biochemical parameter that reflect highly fluctuating GH levels over time [9,10]. However, serum IGF-1 levels also vary with light exposure. Hence, an accurate assessment of the endocrine profile depends on the time of blood collection and the phase of the underlying hormonal rhythm [11].

Astragalus extract mixture HT042 is a standardized functional food granted by the Korean Food and Drug Administration for promoting “Children’s Height Growth”. HT042 is a functional food made *Astragalus* extract mixture and permitted by the Korean Food and Drug Administration. HT042 is a standardized multi-herb mixture composed of the stem of *Eleutherococcus senticosus* and the roots of *Astragalus membranaceus* and *Phlomis umbrosa*. The underlying mechanism of bone growth induced by HT042 involves stimulation of GH secretion, leading to a systemic and local production of IGF-1 [12]. HT042 induces long bone growth by stimulating cartilage cell proliferation and subsequent growth plate hypertrophy in growth plates [13,14]. HT042 has been shown to have various effects such as stimulating bone growth rate, increasing IGF-1 mRNA expression in the proliferation and hypertrophy areas of the growth plate, and increasing the blood concentration of IGF-1 [12,13,14]. Additionally, children of mild short stature who received HT042 supplementation for 12 or 24 weeks showed a significant difference in height growth compared to placebo treated children in two randomized controlled trial [15,16]. In the case of GH injection, which is virtually the only treatment, it is an ideal treatment for short stature with congenital problems. However, while the effects of GH are relatively modest for many children who are not deficient in GH, the risk benefit analysis is controversial due to the burden of daily injections and the very high cost [17,18]. Therefore, it is necessary to reinforce scientific evidences on the efficacy and mechanism studies of natural products that might help growing children through oral intake.

However, there has been no research so far on whether HT042 expresses GH mRNA in the pituitary gland or whether it affects mRNA expression, such as GHRH, growth hormone secretagogue receptor (GHS-R), and SST mRNA expression in the hypothalamus. In addition, we determined whether HT042 had an effect on circulatory IGF-1 after its administration, and the growth effect was also assessed in this study.

## 2. Materials and Methods

### 2.1. Preparation of Sample and Analysis of High Performance Liquid Chromatography (HPLC)

HT042 was obtained from NeuMed Company (Seoul, Korea). Manufacturing process and quality management of HT042 were performed according to the methods outlined by the Korean FDA. Standardized HT042 consists of 0.36% eleuthero-side E, 0.15% shanzhiside methyl ester, and 0.008% formononetin. HPLC analysis was carried out on a Waters Company (Milford, MA, USA) equipped with a pump (Waters 1525), an autosampler (Waters 2707) and a photodiode array detector (Waters 2998). The mobile contained phosphoric acid (0.5%) (A) and acetonitrile (B), starting with 35% solvent B and installing a gradient to obtain formononetin, 35% (0 min), 35% (15 min), 65% (25 min), 35% (28 min), 35% (30 min); shanzhiside methyl ester, and eleuthero-side E, 5% (0 min), 17% (20 min), 22% (30 min), 30% (40 min), 5% (45 min). A reverse-phase SunFire C18 column (250 × 4.6 mm, particle size: 5 μm, Waters) was utilized. The flow rate was 1.0 mL min^−1^, and elution was used and kept at 40 °C. Eleuthero-side E, shanzhiside methyl ester, and formononetin were operated at 210 nm, 235 nm, and 245 nm, respectively.

### 2.2. Animals Housing and Treatment

Male Sprague-Dawley (SD) rats (31 days old, 60 ± 10 g) were supplied by Samtako Co., (Gyeonggi-do, Osan-si, Korea). This study was conducted in accordance with the instructions of the Institutional Animal Care and Use Committee (KISTEM-IACUC-2016-001). Animals’ room was maintained under steady humidity (55 ± 10%), temperature (23 ± 2 °C) and lighting (lights on from 07:00 to 19:00) conditions. Rats were given food and water ad libitum.

After 9-day acclimation, rats were divided into 4 groups: control, recombinant human GH (rhGH, Eutropin, LG Co., Seoul, Korea) 200 μg/kg, HT042 100 mg/kg and HT042 300 mg/kg. The day before sacrifice, HT042 and distilled water control samples were administered orally at 8:00 AM, and rhGH was injected subcutaneously. After 24 h of sample administration, the rats were anesthetized with isoflurane. Blood was gathered and viscera (liver, hypothalamus, pituitary gland) were removed. Hypothalamus was removed 12 h later for detection of GHRH mRNA expression.

### 2.3. Enzyme-Linked Immunosorbent Assay (ELISA)

For quantitative analysis of the serum IGF-1 level, a sandwich assay for IGF-1 concentration was conducted in a 96-well plate in duplicate in accordance with the manufacturer’s instructions (RMEE25R, BioVendor Co., Brno, Czech Republic).

### 2.4. Real-Time Quantitative Polymerase Chain Reaction (RT-qPCR)

After sacrificing the rats, viscera were immediately removed, rinsed, and stored at −80 °C. The total RNA was extracted by QIAzol reagent (Qiagen Co., Valencia, CA, USA) and transformed into cDNA with a transcription kit (Applied Biosystems Co., Foster City, CA, USA) in accordance with the guidelines. qPCR was carried out on a RT-PCR system (Applied Biosystems Co., USA) as follows: at 95 °C for 10 min, at 95 °C for 15 s for 40 cycles and at 60 °C for 60 s. The primers were designed by Bioneer company (Daejeon, Korea). The terms were as follows: at 95 °C for 10 min, at 95 °C for 15 s for 40 cycles and at 60 °C for 60 s. The primer IGF-1, IGFBP-3 and GAPDH are derived from GenBank Accession Number M15481, GenBank Accession Number NM_012588 and GenBank Accession Number NM_017008, respectively. The primer sequences are shown in Table 1.

### 2.5. Longitudinal Bone Growth Rate

Tetracycline staining, a method for observing growth plate length for analysis in bone, was performed on growth plates. 72 h before sacrifice, tetracycline hydrochloride was injected (20 mg/kg, i.p.) [19]. The dissected tibias were fixated with paraformaldehyde (4%), decalcified in ethylenediamine tetra acetic acid (EDTA) solution (50 mM) and dehydrated overnight in sucrose solutions (30%). Dehydrated tibias were cut off from the sagittal plane with a thickness of 40 μm on a cryostat (CM3050S, Leica Microsystems Co., Berlin, Germany). The daily longitudinal growth rate was measured by dividing the distance between chondroosseous junction and tetracycline label into three. Tetracycline label was viewed with a fluorescent microscope (BX50, Olympus, Tokyo, Japan). The distance was measured with Image J by 3 different researchers in a blind manner.

### 2.6. Immunohistochemistry

The sagittal parts were incubated overnight at 4 °C with goat BMP-2 primary-antibody and rabbit IGF-1 (1:200, Santa Cruz, Dallas, TX, USA) after pretreatment [19]. After cleaning, the parts were incubated with rabbit secondary-antibody (1:200, Jackson, West Grove, PA, USA) for 1 h. Subsequent to washing, parts were incubated with avidin-biotin complex reagent (1:100, Vector, Burlingame, CA, USA) for an hour. The parts were stained with a 3,3-diaminobenzidine (0.05%) and hydrogen peroxide.

### 2.7. Statistical Analysis

All data were analyzed by one-way analysis of variance using GraphPad Prism 6 software (GraphPad Software, San Diego, CA, USA). All values were shown as means with standard error (SE). Newman-Keuls test was utilized for multiple comparisons.

## 3. Results

### 3.1. HPLC Analysis of HT042

**Figure 1:**
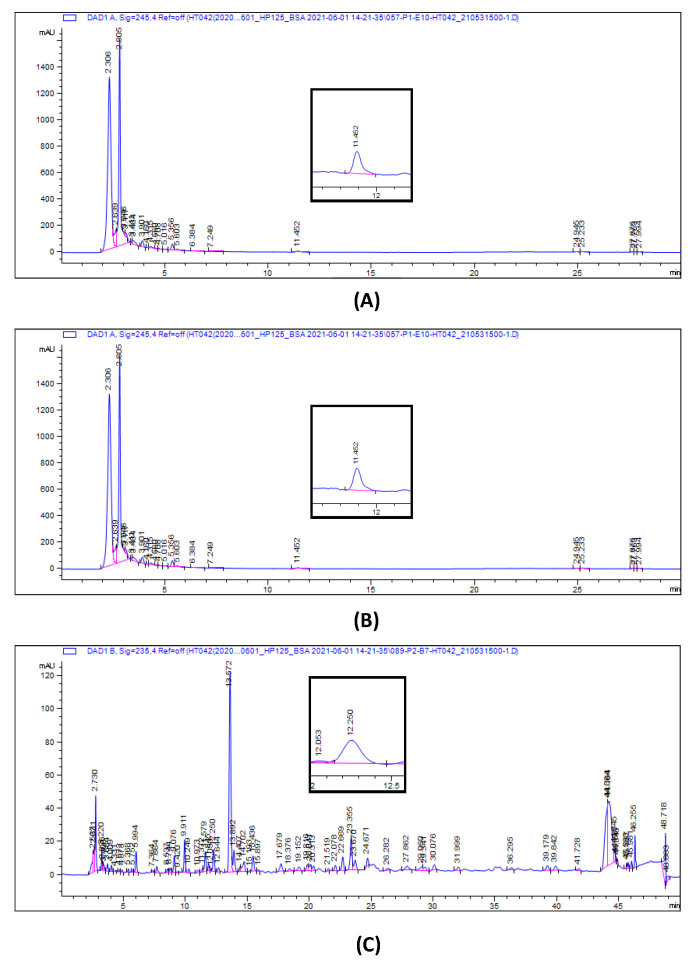
High performance liquid chromatography chromatograms of formononetin (**A**), eleutheroside E (**B**) and shanzhiside methyl ester (**C**) in HT042.

The formononetin, eleuthero-side E, and shanzhiside methyl ester contents of the HT042 were quantified by HPLC (Figure 1). HT042 contains 4.19 ± 0.03 mg/g of formononetin, 1.50 ± 0.05 mg/g of eleuthero-side E, and 0.08 ± 0.00 mg/g of shanzhiside methyl ester. The data were measured in triplicate for reproducibility of the analysis and all values were presented as mean with SE.

### 3.2. Effect on Serum IGF-1 Concentrations

**Figure 2:**
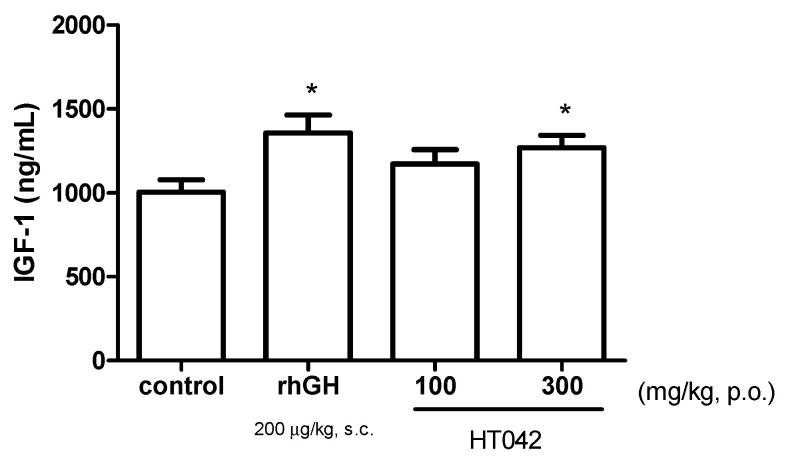
The serum level of insulin-like growth factor-1 estimated using enzyme-linked immunosorbent assay. Data are expressed as mean ± standard error (*n* = 8 per group), * *p* < 0.05.

To estimate the effect of HT042 on serum IGF-1 levels, serum levels were determined 24 h after treatment. When rhGH was administered, the highest was 1358 ± 303.1 ng/mL, but this was 1269 ± 208.7 ng/mL when HT042 300 mg/kg was administered, which was not significantly different (Figure 2).

### 3.3. Effect of HT042 on mRNA Expression of GH, GHRH-R, and GHS-R in the Pituitary

**Figure 3:**
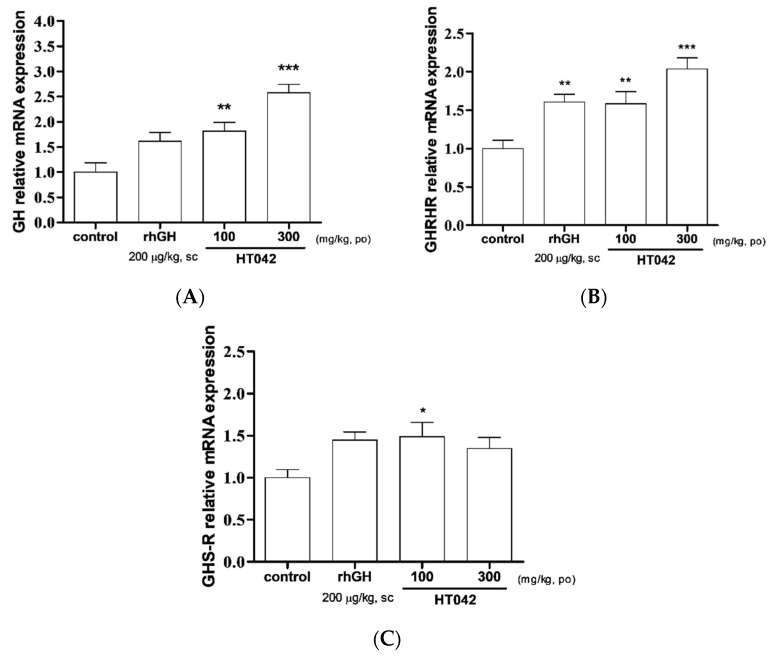
Relative expression of mRNA of (**A**) growth hormone; (**B**) growth hormone-releasing hormone receptor and (**C**) growth hormone secretagogue receptor in the pituitary measured by quantitative real-time quantitative polymerase chain reaction. Data are expressed as mean ± standard error. (*n* = 8 per group), *p < 0.05, ** *p* < 0.01, *** *p* < 0.001.

GH mRNA expression significantly increased 2.4-times in the HT042 300 mg/kg group compared to that in the control (Figure 3A). In comparison with the control, the level of GHRH-R mRNA expression was highest value with administration of 300 mg/kg HT042 (Figure 3B). GHS-R mRNA expression was significantly higher in HT042 100 mg/kg group, while rhGH and HT042 300 mg/kg were showed a tendency to increase compared to control group, but the difference was quasi-significant (Figure 3C).

### 3.4. Effect of HT042 on mRNA Expression of GHRH and SST in the Hypothalamus

**Figure 4:**
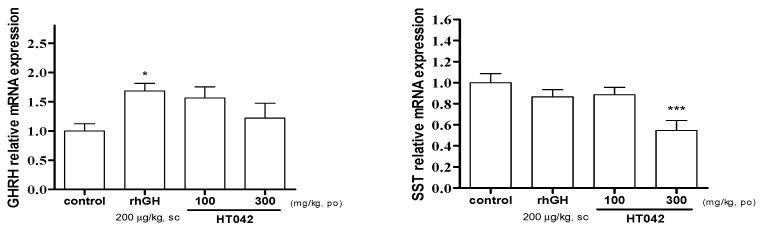
Relative expression of mRNA of growth hormone-releasing hormone and somatostatin in the hypothalamus measured by quantitative real-time quantitative polymerase chain reaction. Data are expressed as mean ± standard error. (*n* = 8 per group), * *p* < 0.05, *** *p* < 0.001.

GHRH mRNA expression was significantly higher in the rhGH group when compared to that in the control, and was higher than that in the treatment group, but was not significantly different (Figure 4). SST mRNA expression was significantly lower at 300 mg/kg HT042 that that of the control.

### 3.5. Effect on IGF-1 and IGFBP-3 mRNA Expression in Liver

**Figure 5:**
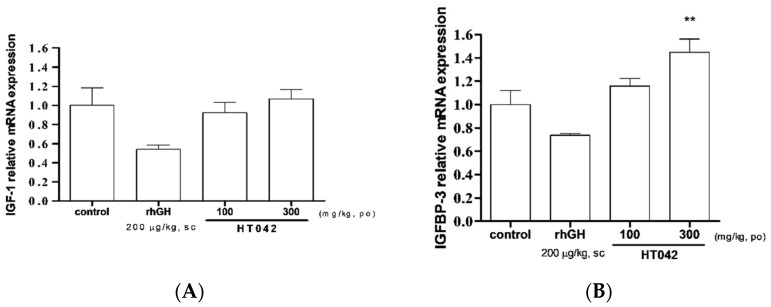
Relative expression of mRNA of (**A**) insulin-like growth factor-1 and (**B**) insulin-like growth factor binding protein-3 in liver measured by quantitative real-time quantitative polymerase chain reaction. Data are expressed as mean ± standard error. (*n* = 8 per group), ** *p* < 0.01.

IGF-1 mRNA expression showed high values at 100 and 300 mg/kg of HT042, but there was no significant difference when compared to those of the control group (Figure 5A). The expression of IGFBP-3 mRNA expression was significantly increased in the 300 mg/kg HT042 group (1.46 times that of the control, Figure 5B).

### 3.6. Effect on Longitudinal Bone Growth Rate

**Figure 6:**
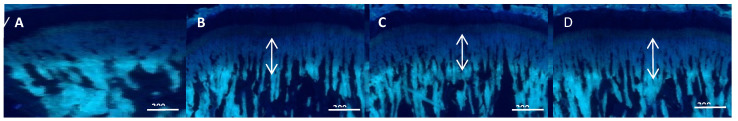
Representative fluorescence photographs of the proximal tibial growth plate in rats. Arrows imply the distance of bone growth in proximal tibia for 72 h period, (**A**) Control; (**B**) rhGH 200 μg/kg (s.c.); (**C**) HT042 100 mg/kg (p.o.); (**D**) HT042 300 mg/kg (p.o.). Scale bar; 200 μm.

**Figure 7:**
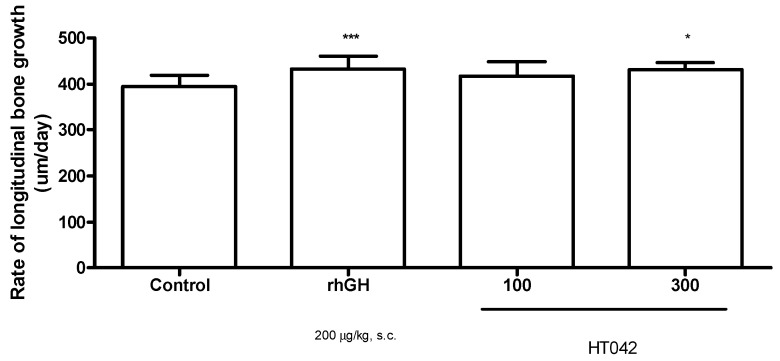
Longitudinal bone growth rate of proximal tibial growth plate of rats measured using fluorescent marker. Data are expressed as mean ± standard error (*n* = 8 per group), * *p* < 0.05, *** *p* < 0.001.

Tetracycline administered to rats formed a fluorescent line (Figure 6). Longitudinal bone growth in the control group was 394.0 ± 24.88 μm/day. rhGH significantly promoted the bone growth rate at 432.3 ± 28.34 μm/day. Bone growth rate with HT042 100 and 300 mg/kg was 416.8 ± 32.12 and 431.4 ± 14.83 mg/kg, respectively. Bone growth rate in the 300 mg/kg of HT042 group was significantly higher than that in the control group (Figure 7).

### 3.7. Effects on IGF-1 and Bone Morphogenetic Protein (BMP)-2 Expression

**Figure 8:**
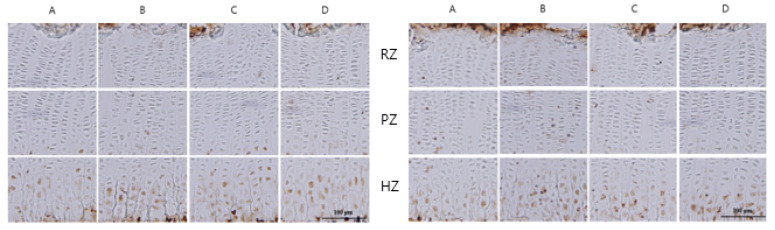
Immunohistochemical localizations of insulin-like growth factor-1 and bone morphogenetic protein-2 on the proximal tibial growth plate in rats. (**A**) control; (**B**) rhGH 200 μg/kg (s.c.); (**C**) HT042 100 mg/kg (p.o.); (**D**) HT042 300 mg/kg (p.o.). RZ; Resting zones, PZ; Proliferative zones, HZ; Hypertrophic zones. Scale bar; 100 μm.

Protein expression of IGF-1 and BMP-2 was performed by antigen-specific immunohistochemical staining in the proximal tibial growth plate. Administration of HT042 or rhGH remarkably increased the intensity of IGF-1 expression in resting zones and hypertrophic zones compared to that in the control. BMP-2 expression was obviously higher in the HT042 or rhGH groups (Figure 8).

## 4. Discussion

Oral administration of *Astragalus* extract mixture HT042 at 100 and 300 mg/kg dose-dependently increased circulatory IGF-1 levels and liver IGF-1 mRNA expression. It was confirmed that GHRH is increased and SST is decreased as the dose of HT042 increased. Oral administration of HT042 has been shown to increase GH mRNA expression levels, as well as GHS-R and GHRH-R mRNA expression levels, and when oral administration was prolonged for 10 days, HT042 increased the growth rate of long bones, and the expression of local IGF-1 and BMP-2 in the growth plate increased compared with that in the control group.

Serum GH level is difficult to accurately evaluate because of its large fluctuations due to pulsatile secretion and short half-life [20]. The concentration of serum IGF-1 is widely known as one of the most accurate marker that reflects the actual GH secretion [21]. Circulating IGF-1 also plays an essential role for sustaining longitudinal growth, as demonstrated in a study showed that liver IGF-1 and ALS double knockout mice were significantly smaller than liver IGF-1 deficient or ALS knockout mice [22]. However, serum IGF-1 level also differ by up to 2.5 times within a day [23], the reason is that cerebellar levels of IGF-1 are dependent to light stimulus [21]. In this study, in order to accurately evaluate whether HT042 affects the GH axis, blood was collected 2 h after the light off, which was a lowest plateau point where the serum IGF-1 level deviation by light was minimized. In addition, since it is known that the time to reach the highest level of IGF-1 is 12–24 h after administration of substances such as GH, HT042 was orally administered to rats 24 h before sacrifice [24,25]. When the experiment was designed in this way, it was confirmed that the serum IGF-1 level was significantly higher at 300 mg/kg HT042 than in the control group. This result suggests that HT042 can increase circulating IGF-1, which is the parameter that reflects GH secretion without huge measurement errors and plays an essential role in longitudinal growth itself. And circulating IGF-1 is synthesized in the liver by GH, administration of HT042 also increased liver IGF-1 and IGFBP-3 mRNA expression. These results also support the suggestion that HT042 administration increases circulating IGF-1, which is consistent with the above result.

In this study, the serum IGF-1 level and liver IGF-1 expression were increased at 24 h after HT042 administration as the time point at which fluctuations were minimized to a plateau. Thus, it is suggested that the growth-stimulating effect of HT042 may be due to the increase in serum IGF-1, which can be stimulated by GH secretion.

GH, GHRH-R, and GHS-R mRNA expression levels in the pituitary were significantly increased in the HT042 group compared to those in the control. Pituitary GH plays an important endocrine part in the expression of multiple somatic and metabolic effects in the entire organic body [26]. GH stimulates the release of IGF-1 from the liver, and this mechanism is responsible for delivering the majority of circulating IGF-1 and longitudinal bone growth. GHRH-R is highly expressed in the pituitary gland, binding to GHRH to stimulate paracrine production of GH [27,28]. Dysfunction of GHRH-R in mice does not normally expand the somatotroph population, leading to hypopituitarism and GH deficiency, resulting in short stature [29]. Other synthetic hexa- or pentapeptides known as GHS (nonpeptidyl GH-secretagogues) also stimulate GH release through specific GHS-R [30,31]. In this study, HT042 modulated the activity of GHRH-R and GHR-S in the pituitary gland and promoted GH secretion, which means that it alters longitudinal growth through GH/IGF-1 mediation.

HT042 increased GHRH mRNA expression and inhibited SST mRNA expression in the hypothalamus. The expression and release of the GH in the pituitary somatic trophozoite are regulated by hypothalamic neuropeptides, such as GHRH and SST [32,33]. After binding to each receptor in somatic trophozoites, these hormones either promote or inhibit the activation of other intracellular messengers and transcription factors, such as Ca^2+^ levels and cAMP reactive element binding required for controlling GH expression and release [34]. Furthermore, it is widely known that IGF-1 stimulates SST and inhibits GHRH expressions in hypothalamus, resulting in GH negative feedback [35,36]. Current data of HT042 promoting IGF-1 secretion but rather increasing GHRH and decreasing SST suggest that HT042 can help GH secretion axis without negative feedback loop through a complex neuroendocrine regulatory system.

When oral administration was prolonged for 10 days, the bone formation rates induced by HT042 at 100 mg/kg and 300 mg/kg were 5.8% and 9.5%, respectively, which indicate an increase compared to that in the control group. Tetracycline is given intraperitoneally, it is accumulated on the newly calcified bone and reveals the bone growth plate length by chelation with calcium [37]. The distance between the cartilage-bone junction and the fluorescent line indicates the rate of bone formation in the cartilage, meaning that the bone length of the growth plate increases within a fixed period of time [38]. Our group made the following advanced experimental design in this study compared to previously published studies to evaluate the effects of growth stimulation within an in vivo model. We used mice of the same age as bone growth rates change excessively with age [39]. To increase the accuracy, we measured the effect of HT042 on endochondral bone formation for 72 h after 10 days of administration, unlike the previous experiment in which HT042 was measured for 48 h 4 days after administration [19,40,41]. We also used sagittal cuts instead of coronal cuts to reduce changes in bony sections. Unlike animal models for disease study, this model needs to be checked for effects in normal rats, so the design has been continuously improved.

HT042 increased the protein expression of local IGF-1 and BMP-2. HT042 highly induced the expression of BMP-2 and IGF-1 in proliferative and hypertrophic regions, reflecting cell proliferation of chondrocytes. Locally expressed IGF-1 is induced primarily by serum GH and binds to the IGF-1 receptor expressed on the chondrocyte surface of the growth plate in the same way as systemic IGF-1. Mice with specific deletion of liver IGF-1 showed comparatively normal growth and development, in spite of 75% reduction in serum IGF-1, indicating that local IGF-1 also make a significant contribution to bone growth [22]. Among other factors affected by GH, BMP is essential for bone tissue formation and induces differentiation and maturation of chondroblasts and chondrocytes [38]. Therefore, BMPs induce the development of epiphyseal growth plates and act as therapeutic agents for bone formation. BMP-2 inhibits osteogenic inhibitors to promote bone formation and stimulates the proliferation and hypertrophy of growth plate chondrocytes, which increases the growth of the longitudinal bone [38,42]. BMP-2 also modulates the action of mitotic IGF-1 in chondrocytes in the epiphyseal plate [43]. With respect to immunohistochemical studies, we suggest that the effect of HT042 can also be due to the upregulation of local IGF-1 and BMP-2 expression in the growth plate, which would be implied a GH-dependent autocrine/paracrine pathway, as well as circulatory IGF-1.

## 5. Conclusions

It was confirmed that the growth-promoting effect of HT042 can be attributed to an increase in serum IGF-1, which can be stimulated by GH secretion. Administration of HT042 modulated the activity of GHRH-R and GHR-S in the pituitary gland and promoted GH secretion, thereby changing longitudinal growth through GH/IGF-1 mediation. Results for GHRH and SST expression showed that the hypothalamus can be influenced and mediated by HT042 through a complex neuroendocrine regulatory system. In addition, it was confirmed that HT042 increased formation in cartilage, which is important for height growth by oral administration for 10 days. Further, immunohistochemical studies revealed that the effect of HT042 could be owing to the upregulation of local IGF-1 and BMP-2 expression in the growth plate, which could be regarded as a GH-dependent autocrine/paracrine pathway, as well as circulatory IGF-1.

## Figures and Tables

**Table 1:** The primer sequences used in the real-time quantitative polymerase chain.

IGF-1	F	5-GCTATGGCTCCAGCATTCG-3
	R	5-TCCGGAAGCAACACTCATCC-3
IGFBP-3	F	5-GGAAAGACGACGTGCATTG-3
	R	5-GCGTATTTGAGCTCCACGTT-3
GAPDH	F	5-TGGCCTCCAAGGAGTAAGAAAC-3
	R	5-CAGCAACTGAGGGCCTCTCT-3

IGF-1–Insulin-like growth factor-1, IGFBP-3–Insulin-like growth factor binding protein-3, GAPDH–Glyceraldehyde 3-phosphate dehydrogenase, F–Forward, R–Reverse.

## Data Availability

Not applicable.
